# Identification of Functional Genes in Pterygium Based on Bioinformatics Analysis

**DOI:** 10.1155/2020/2383516

**Published:** 2020-11-20

**Authors:** Yuting Xu, Chen Qiao, Siying He, Chen Lu, Shiqi Dong, Xiying Wu, Ming Yan, Fang Zheng

**Affiliations:** ^1^Department of Ophthamology, Zhongnan Hospital of Wuhan University, Wuhan, Hubei 430071, China; ^2^Department of Corneal, Hankou Aier Eye Hospital, Wuhan, Hubei 430024, China; ^3^Center for Gene Diagnosis & Core Lab, Zhongnan Hospital of Wuhan University, Wuhan, Hubei 430071, China; ^4^Department of General Surgery, Second Affiliated Hospital, Nanjing Medical University, Nanjing, Jiangsu 210000, China

## Abstract

**Purpose:**

The competing endogenous RNA (ceRNA) network regulatory has been investigated in the occurrence and development of many diseases. This research aimed at identifying the key RNAs of ceRNA network in pterygium and exploring the underlying molecular mechanism.

**Methods:**

Differentially expressed long noncoding RNAs (lncRNAs), microRNAs (miRNAs), and mRNAs were obtained from the Gene Expression Omnibus (GEO) database and analyzed with the R programming language. LncRNA and miRNA expressions were extracted and pooled by the GEO database and compared with those in published literature. The lncRNA-miRNA-mRNA network was constructed of selected lncRNAs, miRNAs, and mRNAs. Metascape was used to perform Gene Ontology (GO) and Kyoto Encyclopedia of Genes and Genomes (KEGG) analyses on mRNAs of the ceRNA network and to perform Protein-Protein Interaction (PPI) Network analysis on the String website to find candidate hub genes. The Comparative Toxicogenomic Database (CTD) was used to find hub genes closely related to pterygium. The differential expressions of hub genes were verified using the reverse transcription-real-time fluorescent quantitative PCR (RT-qPCR).

**Result:**

There were 8 lncRNAs, 12 miRNAs, and 94 mRNAs filtered to construct the primary ceRNA network. A key lncRNA LIN00472 ranking the top 1 node degree was selected to reconstruct the LIN00472 network. The GO and KEGG pathway enrichment showed the mRNAs in ceRNA networks mainly involved in homophilic cell adhesion via plasma membrane adhesion molecules, developmental growth, regulation of neuron projection development, cell maturation, synapse assembly, central nervous system neuron differentiation, and PID FOXM1 PATHWAY. According to the Protein-Protein Interaction Network (PPI) analysis on mRNAs in LINC00472 network, 10 candidate hub genes were identified according to node degree ranking. Using the CTD database, we identified 8 hub genes closely related to pterygium; RT-qPCR verified 6 of them were highly expressed in pterygium.

**Conclusion:**

Our research found LINC00472 might regulate 8 hub miRNAs (miR-29b-3p, miR-183-5p, miR-138-5p, miR-211-5p, miR-221-3p, miR-218-5p, miR-642a-5p, miR-5000-3p) and 6 hub genes (CDH2, MYC, CCNB1, RELN, ERBB4, RB1) in the ceRNA network through mainly PID FOXM1 PATHWAY and play an important role in the development of pterygium.

## 1. Introduction

Pterygium is a common ocular surface disease characterized by a triangular-shaped growth consisting of fibrotic subconjunctival connective tissue and hypertrophy of the overlying conjunctival epithelium [1]. The population affected by pterygium reached 200 million globally, and the prevalence in China was 108.65 million [2, 3]. Pterygium has long been considered as a chronic degenerative condition; however, after abnormal expression of the p53 protein was found in the epithelium [4], pterygium is now considered ultraviolet-related uncontrolled cell proliferation, similar to tumors [5]. The development of pterygium is a complicated process involving cell proliferation, migration, inflammatory infiltrates, fibrosis, angiogenesis, and extracellular matrix breakdown [2]. However, the mechanism of pterygium formation and development is still not completely understood. Studies have demonstrated that noncoding RNAs served crucial roles in numerous diseases [6–8]. They are classified into two classes: small noncoding RNAs (microRNAs (miRNAs), small interfering RNAs, and transfer RNAs) and long noncoding RNAs (lncRNAs). Salmena et al. proposed the competing endogenous RNA (ceRNA) hypothesis, wherein lncRNAs harboring miRNA response elements competed with one another to bind to a common miRNA and thereby acted as molecular “sponges” and depressed the target genes of the miRNAs [9]. The ceRNA network has been demonstrated in numerous diseases, particularly in cancer. By establishing the lncRNA-miRNA-mRNA network, Wang et al. identified functional genes in heart failure and provided further insights into the important roles of the ceRNA network in heart failure [10]. Yang et al. discovered that lncRNA RP11-169F17.1 and RP11-669N7.2 could become novel biomarkers of stomach adenocarcinoma assessed by the construction of the ceRNA network [11]. Zhu et al. reported that 4 lncRNAs might act as potential therapeutic targets or candidate prognostic biomarkers in clear cell kidney carcinoma by reconstruction and comprehensive analysis of the ceRNA regulatory network [12].

However, there is only 1 research on constructing ceRNA networks for pterygium [13]. The hub genes of the network and their roles in the development of pterygium have not been fully understood. Therefore, we collected the lncRNA, miRNA, and mRNA datasets and published literature; filtered out differentially expressed long noncoding RNAs (DELs), microRNAs (DEMis), and mRNAs (DEMs) to constructed ceRNA networks; and identified hub genes closely related to pterygium, based on the functional and pathway analysis on mRNAs in the ceRNA network.

## 2. Materials and Methods

### 2.1. Data Collection

The Gene Expression Omnibus (GEO) is an international, public functional genomics data repository for high-throughput microarray and next-generation sequences [14]. The published pterygium gene expression profiles (GSE83627, GSE21346, GSE51995, and GSE2513) were downloaded [15–19]. The GSE83627 lncRNA data set was collected using GPL14550 platforms (Agilent-028004 SurePrint G3 Human GE 8 × 60 K Microarray, Agilent Technologies, Inc.) and included samples from 4 pterygium and 4 healthy conjunctiva tissues. The miRNA expression data of GSE21346 were based on the GPL7723 platforms (miRCURYLNA microRNA Array, v.11.0-hsa, mmu & rno, Exiqon A/S) and consisted of samples from 3 pterygium and 3 healthy conjunctiva tissues. The mRNA expression data of GSE51995 were from 4 pterygium samples and 4 healthy conjunctiva tissues and were based on GPL14550 platforms (Agilent-028004 SurePrint G3 Human GE 8 × 60 K Microarray, Agilent Technologies, Inc.). Another mRNA expression data of GSE2513 were from 8 pterygium samples and 4 healthy conjunctiva tissues and were based on GPL96 platforms (Affymetrix Human Genome U133A Array, Affymetrix). In order to make the datasets more abundant, we executed a systemic search in PubMed, CNKI, and Web of Science and found 2 microarray studies on DELs and 7 studies on DEMis, including 4 microarray assays studies and 3 experimental verification studies.

### 2.2. Data Preprocessing

All downloaded data sets were standardized through the limma package of R 3.6.3, and the standardized expression matrix was used for the differential expression analysis. The DELs, DEMis, and DEMs between pterygium samples and healthy control conjunctiva tissues were selected according to ∣log_2_foldchange(FC) | ≥1 and *P* values <0.05 [20]. Sequentially, DELs and DEMis were pooled based on the microarray data in the searched studies, respectively, with the Draw Venn Diagram website (http://bioinformatics.psb.ugent.be/webtools/Venn/). And the pooled results were used as candidate DELs and DEMis for the following analysis. The volcano plots and the heatmaps of DELs, DEMis, and DEMs expressions were set up using the ggpolt 2 package and pheatmap package in R 3.6.3 separately.

### 2.3. Prediction of Target lncRNAs and mRNAs of miRNAs

The lncRNA targets of the candidate miRNAs were predicted using DIANA-LncBase v3 (https://diana.e-ce.uth.gr/lncbasev3/interactions), which identifies miRNA and lncRNA interactions derived from manually curated publications and the analysis of 153 AGO CLIP-Seq libraries [21], and starbase 2.0 (http://starbase.sysu.edu.cn/) [22]. The predicted DELs could be selected for ceRNA network construction. Data for miRNA and mRNA interactions were downloaded from miRDB (http://www.mirdb.org/), TargetScanHuman7.1 (http://www.targetscan.org/), and miWalk2.0 (http://zmf.umm.uni-heidelberg.de/apps/zmf/mirwalk2/miRretsys-self.html), and DEMs which appeared in all three databases were used for the following analysis.

### 2.4. Construction of the lncRNA-miRNA-mRNA Network

Based on the associations between lncRNAs-miRNAs and miRNAs-mRNAs, the ceRNA network was constructed. First, the upregulated and downregulated RNAs (lncRNAs, miRNAs, and mRNAs) were assigned ∣log2FC | >1 with *P* values <0.05; then, the lncRNA-miRNA-mRNA network was visualized by using the Cytoscape 3.7.2 software [23], and all node degrees of the ceRNA network were calculated using the CytoHubba plugin [24] of Cytoscape 3.7.2. The significant lncRNA was selected according to the node degree ranking, and a new ceRNA network was reconstructed centered on the top lncRNA.

### 2.5. Functional Analysis on mRNAs in ceRNA Network for Discovering Hub Genes

To analyze the function of mRNAs in ceRNA networks, GO and KEGG analyses were performed by Metascape (http://metascape.org/) [25]. The protein-protein interaction (PPI) network of mRNAs in the ceRNA network was constructed by the STRING database with the confidence score > 0.4 as the cutoff level, providing the critical assessment and integration of protein-protein interactions of genes, including direct (physical) as well as indirect (function) associations [26, 27]. Then, we used the CytoHubba plugin of Cytoscape 3.7.2 to analyze the PPI network and to choose the candidate hub genes with top node degrees. Sequentially, we identified hub genes closely related to pterygium using the Comparative Toxicogenomic Database (CTD), which provides interactions between diseases-genes and diseases-drugs.

### 2.6. Validation of Hub Genes

#### 2.6.1. Patient Sample Preparation

There were 20 primary pterygium specimens obtained in Zhongnan Hospital of Wuhan University during pterygium surgery. The control conjunctival tissues were derived from healthy conjunctival tissues on the temporal side of the surgical eye of the same patient, with a size of 1.5mm × 1.5mm. There were 9 males aged 40-84 (58.89 ± 1.24) years old and 11 females aged 40-79 (59.52 ± 0.86) years old. All participants are of Han nationality and have no blood relationship. This study was reviewed and approved by the Ethics Committee of Zhongnan Hospital of Wuhan University, and the written informed consent of the patients has been obtained before the study. Patients with recurrent pterygium, history of severe eye trauma, eye infection, cataract, glaucoma, acute dacryocystitis, and severe mental illness were excluded.

#### 2.6.2. Tissue RNA Extraction and RT-qPCR

All specimens were collected during the operation and immediately placed in RNA protective agent (RNA later™, SIGMA, USA), stored at -20°C for usage. RNA was extracted from 80 mg specimen tissue using TRIZOL reagent. The relative expression of hub genes was measured using a reverse kit (HiScript® III RT SuperMix for qPCR (+gDNA wiper)) and ChamQ™ Universal SYBR® qPCR Master Mix (Vazyme company, Nanjing, China) on CFX96 fluorescence qPCR instrument (Bio-Rad, USA). And the PCR reaction was carried as follows: predenaturation at 95°C for 30 seconds, denaturation at 95°C for 3-10 seconds, and annealing extension at 60°C for 10-30 seconds, for 40 cycles. Using GAPDH as internal references, the relative expression of genes was calculated by the 2^-△Ct^ method. The primer sequences are listed in Supplementary Table 1.

## 3. Results

### 3.1. Filtered DELs, DEMis, and DEMs

**Figure 1:**
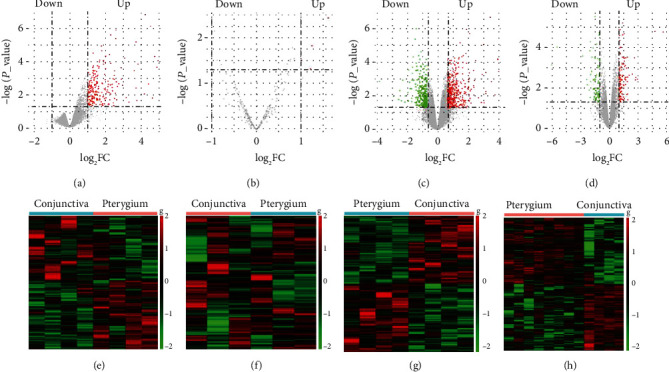
The differentially expressional pattern of RNAs from pterygium and control conjunctiva. (a–d) Volcano plots of DELs, DEMis, and DEMs: (a) DELs (GSE83627); (b) DEMis (GSE21346); (c) DEMs (GSE51995); (d) DEMs (GSE2513). Each point indicates an RNA. Green dots denote the downregulated RNAs, red points represent the upregulated RNAs under the same thresholds, and gray points indicate RNAs that did not change significantly. The criteria are based on ∣log2foldchange | ≥1 and *P* values <0.05. (e–h) Heat maps of DERNAs (e) DELs (GSE83627), (f) DEMis (GSE21346), (g) DEMs (GSE51995), and (h) DEMs (GSE2513). Each column represents one sample, and each row refers to an RNA. The color legend is on the top-right of the figure. Green indicates RNAs with a lower expression relative to the geometrical means; red indicates genes with a greater expression relative to the geometrical means. DELs represent differentially expressed long noncoding RNAs; DEMis represent differentially expressed microRNAs; DEMs represent differentially expressed mRNAs.

**Figure 2:**
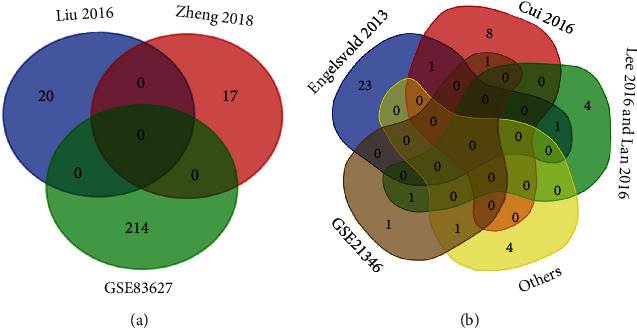
Analysis of the DELs and DEMis from microarray results and 9 publications. (a) In the Venn diagram of two studies and GSE83627, there are no overlapped lncRNAs in the three studies. (b) Venn diagram of GSE21346 and 7 publications of microRNAs. DELs represent differentially expressed long noncoding RNAs; DEMis represent differentially expressed microRNAs.

There are four recruited pterygium datasets from the GEO search, including the lncRNA data set (GSE83627), miRNA data set (GSE21346), and mRNA data sets (GSE51995 and GSE2513). In Figure 1, the expression of DELs, DEMis, and DEMs is shown by volcano plots and heat maps (GSE83627 (Figure 1(e)), GSE21346 (Figure 1(f)), GSE51995 (Figure 1(g)), GSE2513 (Figure 1(h)). After the limma package differential analysis, 214 upregulated lncRNAs were selected (Figure 1(a)), 4 upregulated miRNAs (miR-138-5p, miR-184, miR-642a-5p, miR-1298-5p) were found (Figure 1(b)), and there are 360 downregulated and 537 upregulated mRNAs in GSE51995 (Figure 1(c)) and GSE2513 (Figure 1(d)) datasets. Using online search keywords (lncRNA and pterygium) in PubMed, CNKI, and Web of Science, we found two publications about lncRNA microarray data of pterygium [28, 29]. There were 10 upregulated lncRNAs and 10 downregulated lncRNAs in Liu's study and 10 upregulated lncRNAs and 7 downregulated lncRNAs in Zheng's research (Supplementary Table 2). Then, we used the Venn diagram to make an intersection analysis with lncRNAs in the two publications and previously selected DELs from datasets (Figure 2(a)). So, we merged the lncRNAs from two studies with DELs; then, finally, 251 lncRNAs were obtained to be DELs in the following study, of which 17 are downregulated lncRNAs and 234 upregulated lncRNAs. Moreover, there were seven references including 4 microarray articles and 3 experimental validation (Supplementary Table 2). In the 4 microarray articles, Engelsvold et al. [30] found 14 upregulated and 12 downregulated miRNAs; Cui et al. [31] found 10 differently expressed miRNAs, including 1 upregulated and 9 downregulated; Lee et al. [32] found 4 upregulated miRNAs (miR-143-3p, miR-181a-2-3p, miR-377-5p, miR-411-5p); and Lan et al. found 2 upregulated miRNAs in pterygium (miR-138-5p, miRPlus-E1233) [33]. Three experimental validation studies confirmed that miR-218-5p was downregulated, and miR-182-5p, miR-183-5p, miR-184, and miR-221-3p were upregulated [34–36]. The Venn diagram was used to remove the duplicate miRNAs and combine the reported miRNA with 4 DEMis filtered in GSE21346 (Figure 2(b)). Then, totally, 46 differentially expressed miRNAs were obtained, among which 21 miRNAs were downregulated and 25 miRNAs were upregulated. Eventually, a total of 360 downregulated mRNAs and 537 upregulated mRNAs of two datasets in GEO were filtered. In a word, we got 251 lncRNAs, 46 miRNAs, and 897 mRNAs as candidate RNAs for subsequent study.

### 3.2. miRNA Predicted lncRNA and mRNA Targets

Firstly, the associations between the DELs and DEMis were assessed to reveal the 12 miRNAs targeting 8 lncRNAs. Subsequently, 94 mRNAs targeted by 12 miRNAs were identified. Finally, the 8 coexpressed lncRNAs, 12 coexpressed miRNAs, and 94 coexpressed mRNAs were selected to construct the primary ceRNA network (Table 1).

### 3.3. lncRNA-miRNA-mRNA Networks

**Figure 3:**
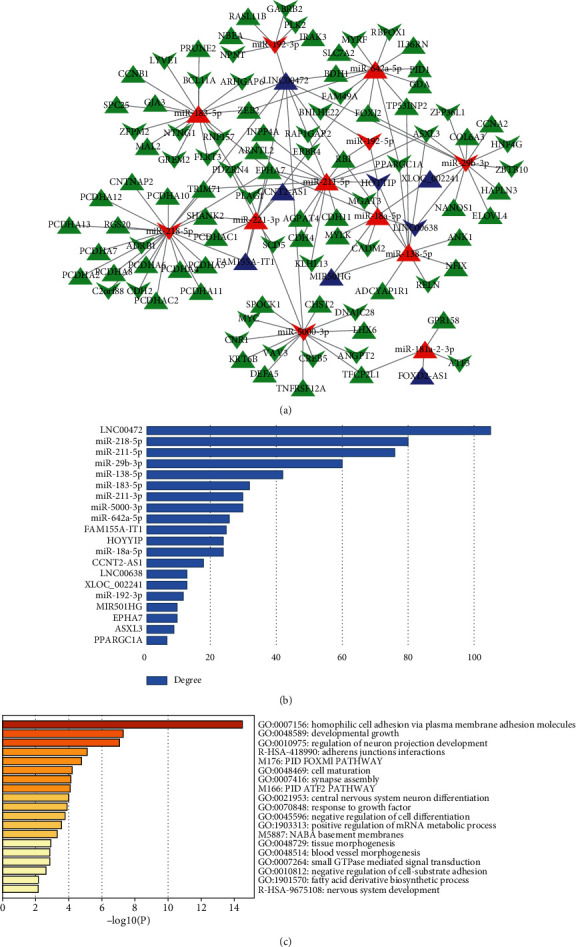
Construction of the primary lncRNA-miRNA-mRNA network and enrichment analysis of the network. (a) The overview of the whole ceRNA network. This interaction network contains 8 lncRNAs, 12 miRNAs, and 94 mRNAs. The regulatory relationship among lncRNA, miRNA, and mRNA was visualized using Cytoscape 3.7.2. (b) The top 20 mRNA node degrees of the primary ceRNA network visualize with a bar chart colored. (c) The top 19 enriched GO and KEGG pathway terms visualize with a bar chart colored. Purple denoted lncRNAs, red denoted miRNAs, and green denoted mRNAs. Triangles denoted upregulated RNAs, and polygons denoted downregulated RNAs.

**Figure 4:**
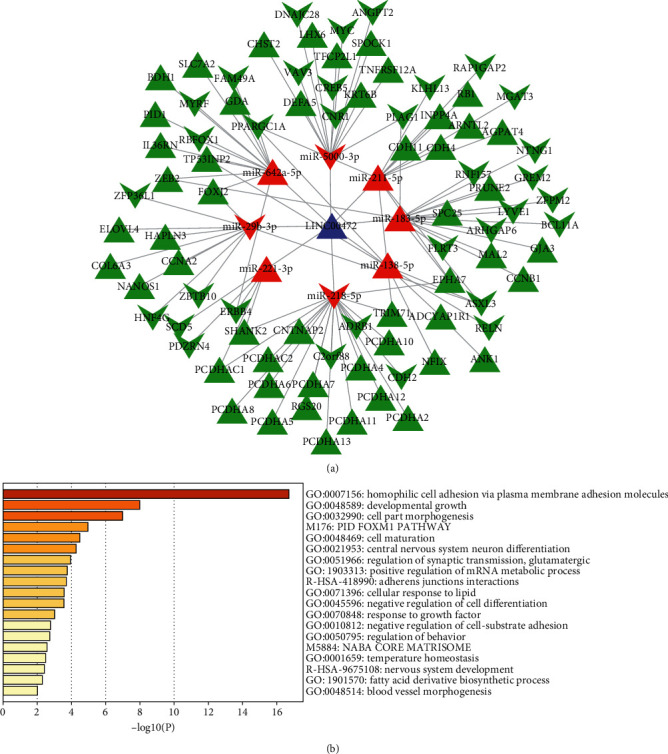
LINC00472 network and enrichment analysis of the network. (a) LINC00472 network. This interaction network contains 1 lncRNA, 8 miRNAs, and 84 mRNAs. (b) The top 19 enriched terms visualized with a bar chart colored. Purple denoted lncRNAs, red denoted miRNAs, and green denoted mRNAs. Triangles denoted upregulated RNAs, and polygons denoted downregulated RNAs.

The lncRNA-miRNA-mRNA ceRNA network was constructed by Cytoscape 3.7.2 (Figure 3(a)), which consisted of above 8 lncRNA nodes, 12 miRNA nodes, 94 mRNA nodes, and 136 edges. According to the node degree ranking, we selected the highest node degree RNA (LINC00472) as the center to construct the LINC00472 network (Figure 3(b)). The new ceRNA network centered by LINC00472 was constructed of 1 lncRNA nodes, 8 miRNA nodes, 84 mRNA nodes, and 106 edges (Figure 4(a)). This suggested that LINC00472 might be a key lncRNA in pterygium functioning through reacting with the 8 miRNAs (miR-29b-3p, miR-183-5p, miR-138-5p, miR-211-5p, miR-221-3p, miR-218-5p, miR-642a-5p, miR-5000-3p). Among them, miRNA-221-3p [34], miR-183-5p [35], and miR-218-5p [36] have been reported to have important function in pterygium. The other 5 miRNAs have been reported to play an important role in cancers. For example, Drummond et al. found miR-29b-3p was involved in a novel mechanism wherein cigarette smoke promoted accelerated cardiac and renal tissue injury in chronic kidney diseases [37]. And miR-138-5p can inhibit the malignant progression of prostate cancer [38] and lung cancer growth, through the miR-138-5p/FOXC1 pathway [39]. miR-211-5p has been confirmed to be related to a variety of cancers, such as cervical cancer [40], the nonsmall cell lung cancer cell [41], breast cancer [42], oral squamous cell carcinoma [43], and papillary thyroid cancer [44]. miR-642a-5p and miR-5000-3p were found have important effects on colon cancer [45, 46]. As we know, pterygium is considered as uncontrolled cell proliferation and is similar to tumor [5], which supports that these miRNAs could also play an important role in the development of pterygium.

### 3.4. Functional Analysis of mRNAs Related to Pterygium

The 94 mRNAs in the primary ceRNA network was investigated by the GO and KEGG pathway enrichment analysis with Metascape. Metascape results were dominated by functional categories, including regulation of homophilic cell adhesion via plasma membrane adhesion molecules, developmental growth, regulation of neuron projection development, adherens junction interactions, cell maturation, synapse assembly, central nervous system neuron differentiation, positive regulation of mRNA metabolic process, negative regulation of cell-substrate adhesion, tissue morphogenesis, and blood vessel morphogenesis. Two pathways were enriched, PID FOXM1 PATHWAY and PID ATF2 PATHWAY. The occurrence of pterygium is closely related to the epithelial-mesenchymal transitions (EMT) and angiogenesis under the tissue [47]. FOXM1 is a member of the forkhead box (FOX) transcription factor family. Previous reports have demonstrated that FOXM1 is oncogenic, in breast cancer [48], hepatocellular carcinoma [49], prostate cancer [50], lung cancer [51], and colorectal cancer [52], and plays important roles in angiogenesis, invasion, and metastasis [53]. It may indicate the mRNAs in the ceRNA network are the biogenesis of the transformation of conjunctival cells into fibroblasts and the formation of subconjunctival neovascularization according to these biological processes and pathways, especially by regulating cell maturation, tissue morphogenesis, blood vessel morphogenesis, and PID FOXM1 PATHWAY (Figure 3(c)).

In order to explore the role of LINC00472 in pterygium, we performed GO and KEGG enrichment analysis again on the 84 mRNAs in the LINC00472 ceRNA network. We found that these genes were enriched in the same biology as the primary ceRNA network, but also in the cell part morphogenesis, regulation of synaptic transmission, glutamatergic, cellular response to lipid, negative regulation of cell differentiation, nervous system development, temperature homeostasis, NABA CORE MATRISOME, regulation of behavior, and response to growth factor. In addition, the mRNAs in both networks are enriched in PID FOXM1 PATHWAY, indicating that LINC00472 is likely to regulate the growth of pterygium through PID FOXM1 PATHWAY (Figure 4(b)).

### 3.5. PPI Network Construction and Analysis of Hub Genes

**Figure 5:**
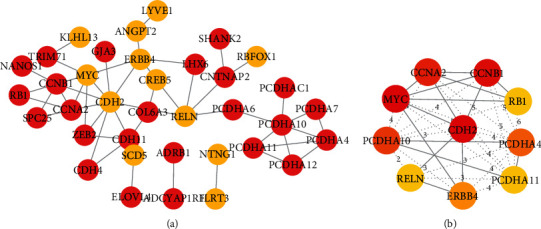
PPI network and hub genes. (a) The whole PPI network of mRNAs in LINC00472 network. Red nodes denoted upregulated genes, and light brown nodes denoted upregulated genes; the lines represented an interaction relationship between the nodes. (b) The top 10 genes evaluated by connectivity degree in the PPI network; the color from red to yellow represented the connectivity degree from high to low.

The PPI network of mRNAs in the LINC00472 network was obtained by using the STRING database, including 36 nodes and 48 edges, and the nodes contain 24 downregulated genes and 12 upregulated genes (Figure 5(a)). The top 10 genes evaluated by connectivity degree in the PPI network were present in CytoHubba, and the result showed that CDH2 was the most significant genes with a connectivity degree of 10, followed by MYC, CCNB1, PCDHA10, RELN, CCNA2, ERBB4, CDH11, PCDHA, and RB1 (Figure 5(b)). We defined the 10 genes as candidate hub genes in the PPI network. It means LINC00472 may regulate pterygium through these 10 key genes.

### 3.6. Identified Hub Genes Closely Related to Pterygium

**Figure 6:**
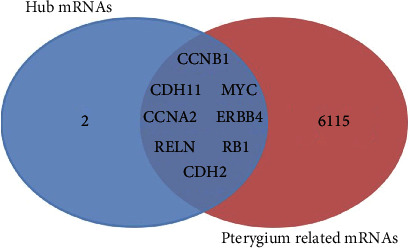
Identified 8 hub genes in the ceRNA network from the Comparative Toxicogenomic Database.

We searched for genes related to pterygium in the CTD and found 6163 mRNAs, these genes are associated with pterygium or its descendants. A gene has either a curated association to the disease (M marker/mechanism and/or T therapeutic) or an inferred association via a curated chemical interaction. Then, we found 8 overlapped genes in the database, CCNB1, CDH11, MYC, CCNA2, ERBB4, RELN, RB1, and CDH2 (Figure 6), as selected hub genes. It implied that the 8 genes in the LINC00472 network may play a key role in pterygium.

### 3.7. Hub Genes Were Verified

**Figure 7:**
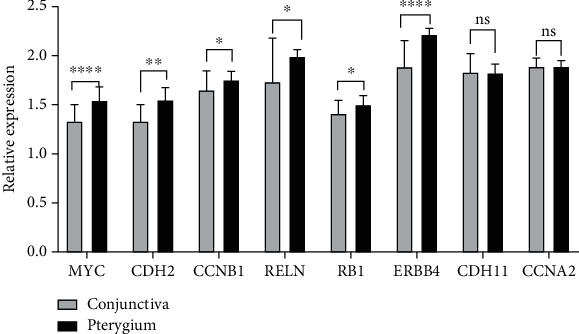
Differential expression of mRNAs between pterygium and paired adjacent control conjunctival tissues was validated by RT-qPCR. ns (no significant difference, *P* > 0.05), ^∗^*P* < 0.05, ^∗∗^*P* < 0.01, ^∗∗∗∗^*P* < 0.0001.

Through RT-qPCR, we used 20 groups of pterygium and control conjunctival tissues to verify the above 8 hub genes. There are 6 genes that are highly expressed in pterygium, MYC, CDH2, CCNB1, RELN, RB1, and ERBB4. Compared with the control conjunctiva, only the expression of CDH11 and CCNA2 showed no significant differences with pterygium (Figure 7). The results indicate that the prediction of the hub genes is reasonably reliable and could be used to discover more hub genes in pterygium.

## 4. Discussion

Pterygium is a common disease in ophthalmology, and its occurrence is related to the proliferation of subconjunctival fibrous tissue and the formation of new blood vessels [15]. The treatment of pterygium is mainly surgical treatment, but the high postoperative recurrence rate is still a problem that is currently difficult to solve [54]. Therefore, effective drug therapy combined with the operation to reduce the recurrence rate of pterygium has become a research hot spot. Recently, with the development of high-throughput genomic platforms and bioinformatic analysis, more and more noncoding RNAs, especially miRNAs and lncRNAs, have been discovered. They serve an important role in the regulation of multiple biological processes, including the development of pterygium [55, 56].

In this study, we mined pterygium-associated RNA expression datasets from the GEO to construct the ceRNA network. Through the analysis of the 4 data sets and 9 publications about lncRNAs and miRNAs, we obtained 251 DELs, 46 DEMis, and 897 DEMs. By the link of miRNA, the primary ceRNA network of pterygium was constructed with 8 lncRNA nodes, 12 miRNA nodes, 94 mRNA nodes, and 136 edges. Since LINC00472 has the highest node degree value of all lncRNAs in the ceRNA network (Figure 3(b)), we took LINC00472 as the center and built the LINC00472 network. And we found genes in the primary ceRNA network mainly enriched in homophilic cell adhesion via plasma membrane adhesion molecules, developmental growth, regulation of neuron projection development, adherens junctions interactions, cell maturation, synapse assembly, central nervous system neuron differentiation, positive regulation of mRNA metabolic process, negative regulation of cell-substrate adhesion, tissue morphogenesis, blood vessel morphogenesis, and PID FOXM1 PATHWAY, while genes in the LINC00472 network enriched in homophilic cell adhesion via plasma membrane adhesion molecules, developmental growth, cell part morphogenesis, regulation of synaptic transmission, glutamatergic, cellular response to lipid, negative regulation of cell differentiation, nervous system development, and temperature homeostasis. In addition, the genes in both networks enriched in PID FOXM1 PAYHWAY, which represents cell proliferation [57], angiogenesis, invasion, and metastasis [53]. This indicated that LINC00472 might play an important role in pterygium through FOXM1 PATHWAY. After the PPI network analysis on mRNAs in the LINC00472 network was visualized by Cytoscape 3.7.2, 10 candidate hub genes were found out. We searched the 10 hub genes in the CTD; 8 hub genes were identified to be involved in pterygium. They are CCNB1, CDH11, MYC, CCNA2, ERBB4, RELN, RB1, and CDH2, and 6 of them were confirmed highly expressed in pterygium by wet experiments (CCNB1, MYC, ERBB4, RELN, RB1, and CDH2). We presume that the LINC00472 network might function in pterygium via the FOXM1 PATHWAY, especially through the regulation of CCNB1, MYC, ERBB4, RELN, RB1, and CDH2 in the ceRNA network. And the data mining based on the public data and publications in the present study is useful and reliable for digging emerging therapy targets of pterygium.

## 5. Conclusion

The present study increased the understanding of the ceRNA-associated regulatory mechanism in the pterygium and identified LINC00472 as a key lncRNA in the whole ceRNA network, which might be highly related to pterygium via the FOXM1 PATHWAY in the LINC00472 network, especially by regulating its related 6 hub genes (CCNB1, MYC, ERBB4, RELN, RB1, and CDH2).

## Figures and Tables

**Table 1:** The RNAs were selected to construct the primary ceRNA network.

RNAs	Gene symbol
lncRNAs	FOXD2-AS1 MIR503HG XLOC_002241 LINC00472 CCNT2-AS1 FAM155A-IT1 LINC00638 HOTTIP
miRNAs	miR-181a-2-3p miR-18a-5p miR-183-5p miR-138-5p miR-211-5p miR-221-3p
	miR-642a-5p miR-29b-3p miR-192-3p miR-218-5p miR-5000-3p miR-192-5p
mRNAs	ADCYAP1R1 ADRB1 AGPAT4 ANK1 ARNTL2 BDH1 CCNA2 CCNB1 CDH11 CDH4 CHST2 CNTNAP2 COL6A3 DEFA5 ELOVL4 EPHA7 FOXJ2 GDAGJA3GPR158 HAPLN3 IL36RN INPP4A IRAK3 KRT6B LHX6 MAL2 MYLK NANOS1 NBEA NFIX PCDHA11 PCDHA12 PCDHA13 PCDHA2 PCDHA4 PCDHA5 PCDHA6 PCDHA7 PCDHA8 PCDHAC1 PCDHAC2 PID1 PRUNE2 RASL11B RB1 RGS20 SHANK2 SLC7A2 SPC25 SPOCK1 TFCP2L1 TNFRSF12A TP53INP2 TRIM71 ZEB2 ADRB1 ANGPT2 ARHGAP6 ASXL3 ATF3 BCL11A BHLHE22 C2orf88 CADM2 CDH2 CNR1 CREB5 DNAJC28 ERBB4 FAM49A FLRT3 GABRB2 GREM2 HNF4G KLHL13 LYVE1 MGAT3 MYC MYRF NPNT NTNG1 PDZRN4 PLAG1 PLK2 PPARGC1A RAP1GAP2 RBFOX1 RELN RNF157 SCD5 VAV3 ZBTB10 ZFP36L1 ZFPM2

## Data Availability

The microarray data (GSE83627, GSE21346, GSE51995, GSE2513) are obtained from the GEO database.

## Supplementary Materials

Supplementary Materials Supplementary Table 1: Primers used for RT-qPCR of hub genes. Supplementary Table 2: Differentially expressed lncRNA and miRNAs in pterygium from 9 publications [28–36].

## References

[1] Chui J., Girolamo N. D., Wakefield D., Coroneo M. T. (2008). The pathogenesis of pterygium: current concepts and their therapeutic implications. *The Ocular Surface*.

[2] Fuest M., Liu Y. C., Coroneo M. T., Mehta J. S. (2017). Femtosecond laser assisted pterygium surgery. *Cornea*.

[3] Song P., Chang X., Wang M., An L., Pan C. (2017). Variations of pterygium prevalence by age, gender and geographic characteristics in China: A systematic review and meta-analysis. *PLoS One*.

[4] Kim K. W., Park S. H., Kim J. C. (2016). Fibroblast biology in pterygia. *Experimental Eye Research*.

[5] Tan D. T., Tang W. Y., Liu Y. P., Goh H. S., Smith D. R. (2000). Apoptosis and apoptosis related gene expression in normal conjunctiva and pterygium. *The British Journal of Ophthalmology*.

[6] Xu S., Kamato D., Little P. J., Nakagawa S., Pelisek J., Jin Z. G. (2019). Targeting epigenetics and non-coding RNAs in atherosclerosis: from mechanisms to therapeutics. *Pharmacology & Therapeutics*.

[7] Sallam T., Sandhu J., Tontonoz P. (2018). Long noncoding RNA discovery in cardiovascular disease: decoding form to function. *Circulation Research*.

[8] Wong C. M., Tsang F. H. C., Ng I. O. L. (2018). Non-coding RNAs in hepatocellular carcinoma: molecular functions and pathological implications. *Nature Reviews Gastroenterology & Hepatology*.

[9] Salmena L., Poliseno L., Tay Y., Kats L., Pandolfi P. P. (2011). A ceRNA hypothesis: the Rosetta Stone of a hidden RNA language?. *Cell*.

[10] Wang G., Zheng X., Zheng Y. (2019). Construction and analysis of the lncRNA-miRNA-mRNA network based on competitive endogenous RNA reveals functional genes in heart failure. *Molecular Medicine Reports*.

[11] Yang J., Song H. (2019). Identification of long noncoding RNA RP11-169F17.1 and RP11-669N7.2 as novel prognostic biomarkers of stomach adenocarcinoma based on integrated bioinformatics analysis. *Epigenomics*.

[12] Zhu H., Lu J., Zhao H. (2018). Functional long noncoding RNAs (lncRNAs) in clear cell kidney carcinoma revealed by reconstruction and comprehensive analysis of the lncRNA-miRNA-mRNA regulatory network. *Medical Science Monitor*.

[13] Xu N., Cui Y., Dong J., Huang L. (2020). Exploring the molecular mechanisms of pterygium by constructing lncRNA-miRNA-mRNA regulatory network. *Investigative Opthalmology & Visual Science*.

[14] Barrett T., Wilhite S. E., Ledoux P. (2013). NCBI GEO: archive for functional genomics data sets--update. *Nucleic Acids Research*.

[15] Lan W., Hou A., Lakshminarayanan R., Lim Y. P., Tong L. (2018). Linc-9432 is a novel pterygium lincRNA which regulates differentiation of fibroblasts. *FEBS Letters*.

[16] Hou A., Lan W., Law K. P. (2014). Evaluation of global differential gene and protein expression in primary pterygium: S100A8 and S100A9 as possible drivers of a signaling network. *PLoS One*.

[17] Riau A. K., Wong T. T., Lan W. (2011). Aberrant DNA methylation of matrix remodeling and cell adhesion related genes in pterygium. *PLoS One*.

[18] Wong Y. W., Chew J., Yang H., Tan D. T., Beuerman R. (2006). Expression of insulin-like growth factor binding protein-3 in pterygium tissue. *The British Journal of Ophthalmology*.

[19] He S., Sun H., Huang Y. (2019). Identification and interaction analysis of significant genes and MicroRNAs in pterygium. *BioMed Research International*.

[20] Ritchie M. E., Phipson B., Wu D. (2015). limma powers differential expression analyses for RNA-sequencing and microarray studies. *Nucleic Acids Research*.

[21] Paraskevopoulou M. D., Vlachos I. S., Karagkouni D. (2016). DIANA-LncBase v2: indexing microRNA targets on non-coding transcripts. *Nucleic Acids Research*.

[22] Li J. H., Liu S., Zhou H., Qu L. H., Yang J. H. (2013). starBase v2.0: decoding miRNA-ceRNA, miRNA-ncRNA and protein–RNA interaction networks from large-scale CLIP-Seq data. *Nucleic Acids Research*.

[23] Shannon P., Markiel A., Ozier O. (2003). Cytoscape: a software environment for integrated models of biomolecular interaction networks. *Genome Research*.

[24] Chin C. H., Chen S. H., Wu H. H., Ho C. W., Ko M. T., Lin C. Y. (2014). cytoHubba: identifying hub objects and sub-networks from complex interactome. *BMC Systems Biology*.

[25] Zhou Y., Zhou B., Pache L. (2019). Metascape provides a biologist-oriented resource for the analysis of systems-level datasets. *Nature Communications*.

[26] Szklarczyk D., Franceschini A., Wyder S. (2015). STRING v10: protein-protein interaction networks, integrated over the tree of life. *Nucleic Acids Research*.

[27] Li M. X., Jin L. T., Wang T. J. (2018). Identification of potential core genes in triple negative breast cancer using bioinformatics analysis. *Oncotargets and Therapy*.

[28] Liu J., Ding X., Yuan L., Zhang X. (2016). Identification of pterygium-related long non-coding RNAs and expression profiling by microarray analysis. *International Journal of Molecular Medicine*.

[29] Zheng J. (2018). *Study on the differential expression profile of lncRNA in pterygium*.

[30] Engelsvold D. H., Utheim T. P., Olstad O. K. (2013). miRNA and mRNA expression profiling identifies members of the miR-200 family as potential regulators of epithelial–mesenchymal transition in pterygium. *Experimental Eye Research*.

[31] Cui Y. H., Li H. Y., Gao Z. X. (2016). Regulation of apoptosis by miR-122 in pterygium via targeting Bcl-w. *Investigative Ophthalmology & Visual Science*.

[32] Lee J. H., Jung S. A., Kwon Y. A., Chung J. L., Kim U. S. (2016). Expression of microRNAs in fibroblast of pterygium. *International Journal of Ophthalmology*.

[33] Lan W., Chen S., Tong L. (2015). MicroRNA-215 regulates fibroblast function: insights from a human fibrotic disease. *Cell Cycle*.

[34] Wu C. W., Cheng Y. W., Hsu N. Y. (2014). MiRNA-221 negatively regulated downstream p27Kip1 gene expression involvement in pterygium pathogenesis. *Molecular Vision*.

[35] İçme G., Yilmaz A., Dinç E., Görür A., Fidanci Ş. B., Tamer L. (2019). Assessment of miR-182, miR-183, miR-184, and miR-221 expressions in primary pterygium and comparison with the normal conjunctiva. *Eye & Contact Lens: Science & Clinical Practice*.

[36] Han S., Chen Y., Gao Y., Sun B., Kong Y. (2019). MicroRNA-218-5p inhibit the migration and proliferation of pterygium epithelial cells by targeting EGFR via PI3K/Akt/mTOR signaling pathway. *Experimental Eye Research*.

[37] Drummond C. A., Crotty Alexander L. E., Haller S. T. (2016). Cigarette smoking causes epigenetic changes associated with cardiorenal fibrosis. *Physiological Genomics*.

[38] Zhang D., Liu X., Zhang Q., Chen X. (2020). miR-138-5p inhibits the malignant progression of prostate cancer by targeting FOXC1. *Cancer Cell International*.

[39] Bai X., Shao J., Zhou S. (2019). Inhibition of lung cancer growth and metastasis by DHA and its metabolite, RvD1, through miR-138-5p/FOXC1 pathway. *Journal of Experimental & Clinical Cancer Research*.

[40] Bai Y., Li X. (2020). hsa_circ_0008285 facilitates the progression of cervical cancer by targeting miR-211-5p/SOX4 axis. *Cancer Management and Research*.

[41] Kang M., Shi J., Li B., Luo M., Xu S., Liu X. (2019). LncRNA DGCR5 regulates the non-small cell lung cancer cell growth, migration, and invasion through regulating miR-211-5p/EPHB6 axis. *BioFactors*.

[42] Yarahmadi S., Abdolvahabi Z., Hesari Z. (2019). Inhibition of sirtuin 1 deacetylase by miR-211-5p provides a mechanism for the induction of cell death in breast cancer cells. *Gene*.

[43] Guo Y., Chen Y., Liu H., Yan W. (2020). Alpinetin inhibits oral squamous cell carcinoma proliferation via miR-211-5p upregulation and notch pathway deactivation. *Nutrition and Cancer*.

[44] Liang M., Jia J., Chen L. (2019). LncRNA MCM3AP-AS1 promotes proliferation and invasion through regulating miR-211-5p/SPARC axis in papillary thyroid cancer. *Endocrine*.

[45] Lin C., Zhang Y., Chen Y., Bai Y., Zhang Y. (2019). Long noncoding RNA LINC01234 promotes serine hydroxymethyltransferase 2 expression and proliferation by competitively binding miR-642a-5p in colon cancer. *Cell Death & Disease*.

[46] Xia Z. S., Wang L., Yu T. (2014). MiR-5000-3p, miR-5009-3P and miR-552: potential microRNA biomarkers of side population cells in colon cancer. *Oncology Reports*.

[47] Wu C. W., Peng M. L., Yeh K. T., Tsai Y. Y., Chiang C. C., Cheng Y. W. (2016). Corrigendum to “Inactivation of p53 in pterygium influence miR-200a expression resulting in ZEB1/ZEB2 up-regulation and EMT processing” [Exp. Eye Res. 146 (2016) 206-211]. *Experimental Eye Research*.

[48] Hamurcu Z., Ashour A., Kahraman N., Ozpolat B. (2016). FOXM1 regulates expression of eukaryotic elongation factor 2 kinase and promotes proliferation, invasion and tumorgenesis of human triple negative breast cancer cells. *Oncotarget*.

[49] Egawa M., Yoshida Y., Ogura S. (2017). Increased expression of Forkhead box M1 transcription factor is associated with clinicopathological features and confers a poor prognosis in human hepatocellular carcinoma. *Hepatology Research*.

[50] Liu Y., Liu Y., Yuan B. (2017). FOXM1 promotes the progression of prostate cancer by regulating PSA gene transcription. *Oncotarget*.

[51] Wang Y., Zhang W., Wen L. (2016). FOXM1 confers resistance to gefitinib in lung adenocarcinoma via a MET/AKT-dependent positive feedback loop. *Oncotarget*.

[52] Wang D., Hu G., du Y. (2017). Aberrant activation of hedgehog signaling promotes cell proliferation via the transcriptional activation of forkhead Box M1 in colorectal cancer cells. *Journal of Experimental & Clinical Cancer Research*.

[53] Xie Y., Cui D., Sui L. (2015). Induction of forkhead box M1 (FoxM1) by EGF through ERK signaling pathway promotes trophoblast cell invasion. *Cell and Tissue Research*.

[54] Hacioglu D., Erdol H. (2017). Developments and current approaches in the treatment of pterygium. *International Ophthalmology*.

[55] Yu B., Shan G. (2016). Functions of long noncoding RNAs in the nucleus. *Nucleus*.

[56] Derrien T., Johnson R., Bussotti G. (2012). The GENCODE v7 catalog of human long noncoding RNAs: analysis of their gene structure, evolution, and expression. *Genome Research*.

[57] Huang B., Mu P., Yu Y. (2020). Inhibition of EZH2 and activation of ERR*γ* synergistically suppresses gastric cancer by inhibiting FOXM1 signaling pathway. *Gastric Cancer*.

