# Supplementary material for: Identification of Functional Genes in Pterygium Based on Bioinformatics Analysis
Source: Biomed Res Int. 2020 Nov 20;2020:2383516. doi: 10.1155/2020/2383516 (PMC7704136; doi:10.1155/2020/2383516)
Supplement: Supplementary Materials — Supplementary Table 1: Primers used for RT-qPCR of hub genes. Supplementary Table 2: Differentially expressed lncRNA and miRNAs in pterygium from 9 publications [28–36]. [file 2383516.f1.docx]

**Supplementary table 1: Primers used for RT-qPCR of hub genes**

| Genes | Primers | Length(bp) |
| --- | --- | --- |
| GAPDH | F: GAAGGTGAAGGTCGGAGTC  R: GAAGATGGTGATGGGATTTC | 172 |
| CCNB1 | F: ACCAAAATACCTACTGGGTCGG  R: GCATGAACCGATCAATAATGG | 140 |
| MYC | F: GGCTCCTGGCAAAAGGTCA  R: CTGCGTAGTTGTGCTGATGT | 119 |
| CDH2 | F: GTCAGCAGAAGTTGAAGAAATAGTG  R: GCAAGTTGATTGGAGGGATG | 104 |
| CCNA2 | F: CAGAAAACCATTGGTCCCTC  R: CACTCACTGGCTTTTCATCTTC | 104 |
| RELN | F: CAGGACCAATCCTTGGAAAC  R: GGTCACACTTGCAACCATGT | 114 |
| ERBB4 | F: AGGAGTGAAATTGGACACAGC  R: TCCATCTCGGTATACAAACTGGT | 75 |
| RB1 | F: TGTAATGGCCACATATAGCAGAAGT  R: TAAGAGGACAAGCAGATTCAAGGTG | 299 |
| CDH11 | F: AATGTGGGAACGTCAGTAA  R: TGTCCATGTTGGGTAGGG | 160 |

## **Supplementary** table 2: Differentially expressed lncRNA and miRNAs in pterygium from 9 publications.

| RNAs | Researchers | LncRNAs | Fold Change | Type | P Values | Microchip type |
| --- | --- | --- | --- | --- | --- | --- |
| LncRNA | Liu 2016 [28] | FOXD2-AS1 | 92.7 | Upregulated | P<0.05 | ArrAystar Human LncRNA Microarray V3.0  (12 pterygium tissue -12conjuntiva tissue) |
|  |  | RP11-78F17.1 | 88.8 | Upregulated |  |  |
|  |  | RP11-702F3.4 | 82.0 | Upregulated |  |  |
|  |  | RP5-963E22.4 | 75.1 | Upregulated |  |  |
|  |  | RP11-611E13.2 | 70.6 | Upregulated |  |  |
|  |  | AF196972.9 | 66.0 | Upregulated |  |  |
|  |  | KIAA0664L3 | 63.5 | Upregulated |  |  |
|  |  | LOC283761 | 61.3 | Upregulated |  |  |
|  |  | RP3-416J7.2 | 58.1 | Upregulated |  |  |
|  |  | LOC100130264 | 56.1 | Upregulated |  |  |
|  |  | LINC00638 | -113.4 | Downregulated |  |  |
|  |  | WI2-2373I1.2 | -104.5 | Downregulated |  |  |
|  |  | ARL6IP6 | -89.9 | Downregulated |  |  |
|  |  | CLCA4 | -68.6 | Downregulated |  |  |
|  |  | TECR | -57.5 | Downregulated |  |  |
|  |  | RP11-398J10.2 | -54.9 | Downregulated |  |  |
|  |  | RP11-61L23.2 | -48.1 | Downregulated |  |  |
|  |  | RP11-420A23.1 | -46.2 | Downregulated |  |  |
|  |  | RP11-217B1.2 | -43.4 | Downregulated |  |  |
|  |  | RP3-434O14.8 | -38.9 | Downregulated |  |  |
| LncRNA | Zheng 2018 [29] | FAM155A-IT1 | 18. 1 | Upregulated | P<0.01 | Agilent Microarray  (20 pterygium tissue -20conjuntiva tissue) |
|  |  | MIR503HG | 18. 7 | Upregulated |  |  |
|  |  | AC103881.1 | 17.2 | Upregulated |  |  |
|  |  | LOC157273 | 13.2 | Upregulated |  |  |
|  |  | CCNT2-AS1 | 12. 8 | Upregulated |  |  |
|  |  | AC007750. 5 | 11. 9 | Upregulated |  |  |
|  |  | RP11-930P14.1 | 9. 7 | Upregulated |  |  |
|  |  | LINC00470 | 8. 9 | Upregulated |  |  |
|  |  | RP11-23D24.2 | 8. 2 | Upregulated |  |  |
|  |  | RP11-119D9.1 | 7. 4 | Upregulated |  |  |
|  |  | RP11-485M7.3 | -15.2 | Downregulated |  |  |
|  |  | RP11-523018.5 | -13.1 | Downregulated |  |  |
|  |  | LINC01226 | -12. 1 | Downregulated |  |  |
|  |  | LINC01226 | -11.3 | Downregulated |  |  |
|  |  | MIR7110 | -11.1 | Downregulated |  |  |
|  |  | HOTTIP | -9. 4 | Downregulated |  |  |
|  |  | PR11-108M9.3 | -9. 3 | Downregulated |  |  |
|  |  | RP11-108M9.3 | -8. 9 | Downregulated |  |  |
|  |  | RP11-108M9.3 | -8. 6 | Downregulated |  |  |
|  |  | LINC00629 | -8 .6 | Downregulated |  |  |
| miRNA | Engelsvold 2013 [30] | miR-1246 | 4.5 | Upregulated | 0.001 | GeneChip®  miRNA2.0 Array, Affymetrix  (8pterygium tissue – 8 control tissue) |
|  |  | miR-486-5p | 4.4 | Upregulated | 0.004 |  |
|  |  | miR-451a | 4.1 | Upregulated | 0.010 |  |
|  |  | miR-3172 | 3.4 | Upregulated | 0.009 |  |
|  |  | miR-3175 | 3.3 | Upregulated | <0.001 |  |
|  |  | miR-1308 | 3.2 | Upregulated | 0.02 |  |
|  |  | miR-1972 | 3 | Upregulated | <0.001 |  |
|  |  | miR-143-3p | 2.7 | Upregulated | 0.008 |  |
|  |  | miR-211-5p | 2.7 | Upregulated | 0.03 |  |
|  |  | miR-665 | 2.3 | Upregulated | 0.01 |  |
|  |  | miR-1973 | 2.2 | Upregulated | 0.04 |  |
|  |  | miR-18a-5p | 2.1 | Upregulated | 0.004 |  |
|  |  | miR-143-5p | 2 | Upregulated | 0.006 |  |
|  |  | miR-663b | 2 | Upregulated | 0.02 |  |
|  |  | miR-675-5p | −2.0 | Downregulated | 0.005 |  |
|  |  | miR-200b-3p | −2.1 | Downregulated | 0.002 |  |
|  |  | miR-200a-5p | −2.3 | Downregulated | 0.002 |  |
|  |  | miR-29b-3p | −2.3 | Downregulated | 0.005 |  |
|  |  | miR-200b-5p | −2.3 | Downregulated | <0.001 |  |
|  |  | miR-210-3p | −2.4 | Downregulated | <0.001 |  |
|  |  | miR-141-3p | −2.5 | Downregulated | <0.001 |  |
|  |  | miR-31-5p | −2.6 | Downregulated | 0.02 |  |
|  |  | miR-200a-3p | −2.7 | Downregulated | <0.001 |  |
|  |  | miR-934 | −3.0 | Downregulated | <0.001 |  |
|  |  | miR-375 | −3.7 | Downregulated | 0.03 |  |
| miRNA | Cui2016 [31] | miR-1298-5p | 2.5 | Upregulated | 0.019 | Exiqon miRCURY LNA^TM^ microRNA Array(3 pterygium tissue-3 control tissue) |
|  |  | miR-122-3p | -14.7 | Downregulated | 0.005 |  |
|  |  | miR-122-5p | -7.4 | Downregulated | 0.037 |  |
|  |  | miR-192-3p | -3.3 | Downregulated | 0.006 |  |
|  |  | miR-192-5p | -4.9 | Downregulated | 0.0002 |  |
|  |  | miR-194-5p | -5.7 | Downregulated | 0.015 |  |
|  |  | miR-302f | -2.8 | Downregulated | 0.006 |  |
|  |  | miR-802 | -4.1 | Downregulated | 0.039 |  |
|  |  | miR-1973 | -2.4 | Downregulated | 0.036 |  |
|  |  | miR-5000-3p | -2.5 | Downregulated | 0.047 |  |
| miRNA | Lee 2016 [32] | miR-143-3p | 2.4 | Upregulated |  | GeneChip miRNA3.0 Array, Affymetrix  (pterygium fibroblasts– control fibroblasts) |
|  |  | miR-181a-2-3p | 3.4 | Upregulated |  |  |
|  |  | miR-377-5p | 2.1 | Upregulated |  |  |
|  |  | miR-411-5p | 3.9 | Upregulated |  |  |
| miRNA | Lan 2015 [33] | miR-138-5p | 3.0 | Upregulated | 0.019 | Exiqon miRCURY LNA^TM^ microRNA Array  (4pterygium tissue -4control tissue) |
|  |  | miRPlus-E1233 | 2.4 | Upregulated | 0.019 |  |
| miRNA | Gökhan Içme 2019 [35] | miR-182-5p | 4.4 | Upregulated | <0.0001 | Experiments confirmed |
|  |  | miR-183-5p | 4.2 | Upregulated | 0.01 |  |
|  |  | miR-184 | 3.0 | Upregulated | 0.01 |  |
| miRNA | Han 2019 [36] | miR-218-5p | — | Downregulated | — | Experiments confirmed |
| miRNA | Wu 2014 [34] | miR-221-3p | — | Downregulated | — | Experiments confirmed |
